# Assessing the Relationships between Interdigital Geometry Quality and Inkjet Printing Parameters

**DOI:** 10.3390/mi13010057

**Published:** 2021-12-30

**Authors:** Federico Bertolucci, Nicolò Berdozzi, Lara Rebaioli, Trunal Patil, Rocco Vertechy, Irene Fassi

**Affiliations:** 1 Industrial Engineering Department, University of Bologna, 40136 Bologna, Italy; federico.bertolucci2@unibo.it (F.B.); nicolo.berdozzi2@unibo.it (N.B.); rocco.vertechy@unibo.it (R.V.); 2 Consiglio Nazionale delle Ricerche, Institute of Intelligent Industrial Technologies and Systems for Advanced Manufacturing, 20133 Milan, Italy; trunal.patil@stiima.cnr.it (T.P.); irene.fassi@stiima.cnr.it (I.F.)

**Keywords:** additive manufacturing, inkjet printing, interdigitated electrodes, image processing, Design of Experiment

## Abstract

Drop on demand (DoD) inkjet printing is a high precision, non-contact, and maskless additive manufacturing technique employed in producing high-precision micrometer-scaled geometries allowing free design manufacturing for flexible devices and printed electronics. A lot of studies exist regarding the ink droplet delivery from the nozzle to the substrate and the jet fluid dynamics, but the literature lacks systematic approaches dealing with the relationship between process parameters and geometrical outcome. This study investigates the influence of the main printing parameters (namely, the spacing between subsequent drops deposited on the substrate, the printing speed, and the nozzle temperature) on the accuracy of a representative geometry consisting of two interdigitated comb-shape electrodes. The study objective was achieved thanks to a proper experimental campaign developed according to Design of Experiments (DoE) methodology. The printing process performance was evaluated by suitable geometrical quantities extracted from the acquired images of the printed samples using a MATLAB algorithm. A drop spacing of 140 µm and 170 µm on the two main directions of the printing plane, with a nozzle temperature of 35 °C, resulted as the most appropriate parameter combination for printing the target geometry. No significant influence of the printing speed on the process outcomes was found, thus choosing the highest speed value within the investigated range can increase productivity.

## 1. Introduction

During the last few decades, the combination of computer design and three-dimensional printing techniques took the workflow of manufacturing processes to a substantial change in several science fields such as biology, life science, and robotics. In particular, inkjet printing has been widely used as a high-precision additive manufacturing technique to produce devices such as transducers [1] and sensors [2]. 

Inkjet printing technology can be divided into two subcategories: continuous and drop on demand (DoD). In the former, the creation of ink droplets is constant and allows performing high-speed printing processes, especially for industrial application. In the latter, a single drop is ejected from the nozzle, allowing smaller drop size generation and higher placement accuracy [3]. Although other printing, coating, and casting processes such as screen printing, spin-coating, top–down etching, or blade casting are commonly used, as they offer a low-cost and large covered area results [4], they involve the contact with the sample and often require the use of masks. DoD inkjet printing takes advantage of its contact-free, maskless, digitally controlled operating mode to design micrometer-scaled geometries. The possibility to create flexible electronics through the DoD printing process gives access to a wide employment in applications such as microelectromechanical systems [5,6], dielectric elastomer transducers [7], electro-adhesive devices [8], as well as optical and electric temperature sensors [9]. All these applications need a high-precision technology that works at the micrometer scale and a free geometry capability. 

As emerged in past works [8], the result of the inkjet printing process strongly depends on the material choice and the interaction between ink and substrate. The literature shows a lot of studies regarding the delivery of the ink droplets from the nozzle to the substrate [10,11], as well as the jet fluid dynamics [12,13], but no systematic approach exists dealing with the relationship between process parameters and geometrical outcome. Indeed, the most conventional way to assess the printing quality is by evaluating the ink–substrate interaction relying on the contact angle (CA) measurement [14,15,16], thus allowing an estimation of the surface energy [17]. Moreover, in the literature, there are no findings about an optimal set of parameters in the printing process neither by relying on the Design of Experiments (DoE) methodology [18].

The interdigitated comb geometry, named interdigital geometry, has been widely used to realize electrodes for flexible and stretchable sensors, transducers, and electro-adhesive devices, as it enhances their performances compared to other geometries [19]. In this study, the interdigital geometry is selected as a representative example, since it requires precise printed lines spaced by a constant gap. Therefore, this work proposes a methodology to set a suitable range for relevant printing parameters (i.e., the spacing between subsequent drops deposited on the substrate, the printing speed, and the nozzle temperature) with the aim of ensuring a good accuracy of the printing process output. This objective is achieved thanks to a proper experimental campaign developed according to the DoE methodology. In this study, the printing quality is considered an attribute of the whole printed geometry; hence, the printing process performance analysis is based on geometrical quantities of interest that are extracted from the acquired images of the manufactured samples using a MATLAB algorithm. 

## 2. Materials and Methods

### 2.1. Geometry Design and Manufacturing

**Figure 1:**
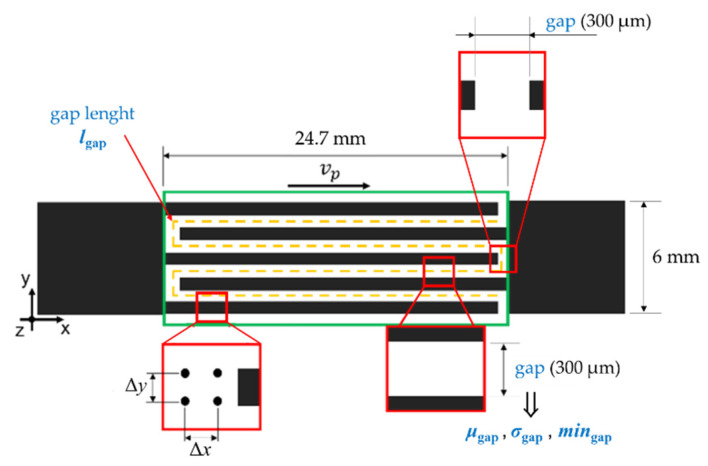
Interdigital geometry.

Figure 1 shows the nominal dimensions of the interdigital geometry selected as reference geometry. The distance between the interdigitated comb shapes is named hereafter “gap”. The line describing the path within the fingers of the comb shapes is named “gap length”.

**Figure 2:**
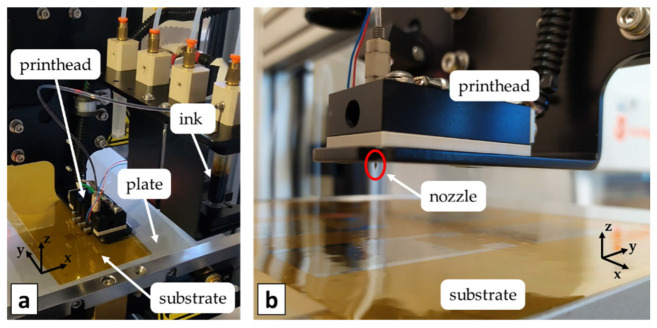
(**a**) MicroFab Jetlab 4xl printer; (**b**) Detail of the printhead with the nozzle.

The MicroFab Jetlab 4xl printer with a 50 μm diameter piezoelectric nozzle is used for ink deposition (Figure 2). An ink drop is generated through a pressure variation in the ink reservoir induced by the vibration of a piezoelectric plate. The drop deposition reference system consists of an aluminum plate sliding horizontally on magnetic rails describing the x-y plane and in a z-axis referred to the motion of the printhead, which is perpendicular to the plate. A vacuum system allows sticking the substrate on the plate during the printing process. A heat control unit is connected both to the printhead and the plate, driving separately the two temperature levels that are sensed by thermocouples.

The interdigital geometry is printed as a combination of several drop arrays. The printing direction corresponds to the main length of the array that is the x-direction (Figure 1). 

A commercial conductive silver-nanoparticle ink (Smart ’Ink S-CS01130 from Genes ’Ink) (Genes Ink 39, avenue Gaston Imbert, Z.I., 13106 Rousset Cedex, France) is used to print due to the high ink stability for droplet formation, good reproducibility of the geometries, and its low resistivity (around 15 µΩ/cm). The S-CS01130 conductive ink features a density of 1 g/mm^3^, a surface tension of 29 dyn/cm, and a dynamic viscosity of 13 cP. Based on the ink rheological properties and the nozzle diameter, the Z number [20,21,22] is equal to 2.93, thus belonging to the printable range according to [23]. The ink is prepared to be printed by filtering it with a 0.45 µm polytetrafluoroethylene (PTFE) syringe filter and 5 min of ultrasonic bath to dissolve any particle aggregation. Once the geometry is printed, the part is cured in an oven at 150 °C for 40 min. 

A 25.4 µm thick polyimide (PI) film is used as substrate. The substrate is industrially produced in rolls by Caplinq (PIT1N/210) (Caplinq, Industrieweg 15E, 1566JN Assendelft, The Netherlands), and it has been preferred to a custom realization to guarantee a homogeneous substrate surface, thus allowing a uniform ink distribution in the printed pattern. No treatment has been performed on the substrate prior to printing to prevent damages due to undesired erosions.

### 2.2. Experimental Design

The effects of the selected parameters on the process performance were studied using a suitable experimental design (Table 1). The four selected factors are the printhead translational speed, or printing speed, along the x-axis (*v*_p_), the subsequent drop spacing along the x-axis (Δ*x*), the subsequent drop spacing along the y-axis (Δ*y*), and the nozzle temperature (*T*_n_). Figure 1 schematically indicates the aforementioned parameters, except for *T*_n_.

**Figure 3:**
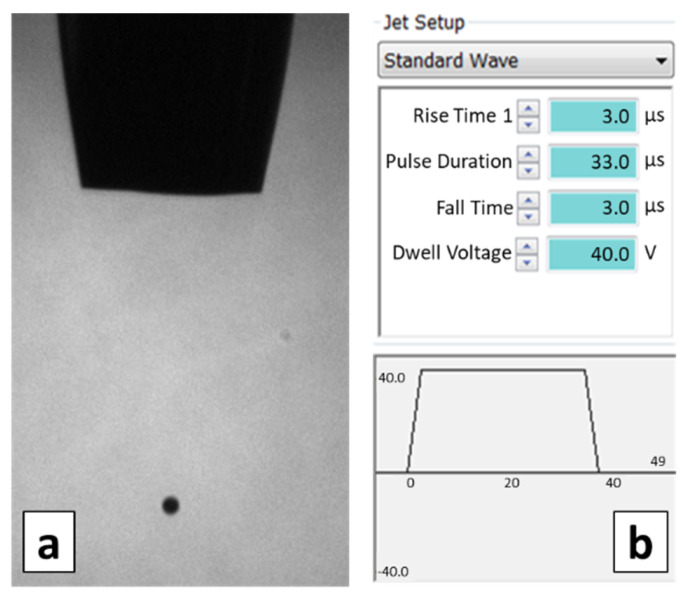
(**a**) Stable drop; (**b**) Voltage waveform.

The printer setup involves the voltage waveform and the backpressure of the printing channel as well as the distance between the nozzle and the printing plate. These parameters are adjusted to obtain a stable drop flight (Figure 3a) [24,25,26] and kept constant throughout the entire experimental design. The printer software allows setting the proper voltage waveform that makes the piezo-crystal oscillate. It allows choosing monopolar and bipolar trapezoidal or sinusoidal waveforms, setting the rise time, the fall time, the wave amplitude (or ‘‘dwell voltage’’), and the pulse duration. In this study, a monopolar trapezoidal wave is chosen, and the set parameter values are shown in Figure 3b. A vacuum system provides the ink reservoir backpressure that is set to −10 Pa, leading to a flat ink meniscus at the nozzle tip, as can be seen in Figure 3a, showing an image captured by an infrared camera and a stroboscopic light system. The nozzle distance from the printing plate is set to 1.5 mm based on previous experiments. 

As shown in Table 1, two levels were selected for each variable factor based on preliminary experiments and in accordance with machine positioning tolerance (i.e., ± 30 µm in the x-y directions), thus resulting in 2^4^ = 16 different experimental conditions. Two replicates were carried out for each experimental condition, while five replicates were carried out for the central point (*v*_p_ = 20 mm/s, Δ*x* = 110 μm and Δ*y* = 140 μm) at each temperature level. Therefore, the whole experimental design included 42 runs, which were completely randomized.

The responses that were analyzed by means of the Analysis of Variance (ANOVA) are the mean value and standard deviation of the gap. Moreover, the minimum value of the gap and the gap length were observed to better assess the printing quality. The calculation of the responses is discussed in Section 2.3.

### 2.3. Measurements and Analysis

A Zeiss Stereo Discovery V20 optical microscope with AxioCam MRc5 digital camera was used to acquire and measure the printed samples. High-resolution images of the entire samples were obtained by stitching multiple tile images acquired at 17.2× magnification and a micron to pixel ratio equal to 3.942. After image acquisition, a MATLAB code was run to extract the geometrical parameters of interest using image processing algorithms.

The geometrical parameters to be evaluated (Figure 1) are defined as follows:

- Mean value of the gap (*μ*_gap_): this parameter represents the average magnitude of the gap;
- Standard deviation of the gap (*σ*_gap_): this parameter shows the regularity of the gap along the shapes;
- Minimum value of the gap (*min*_gap_): this parameter can highlight singularities in the contour lines, such as extra ink deposits, and interconnections between the comb-shapes;
- Gap length (*l*_gap_): this parameter can show if there are some disconnections within the comb shapes.

**Figure 4:**
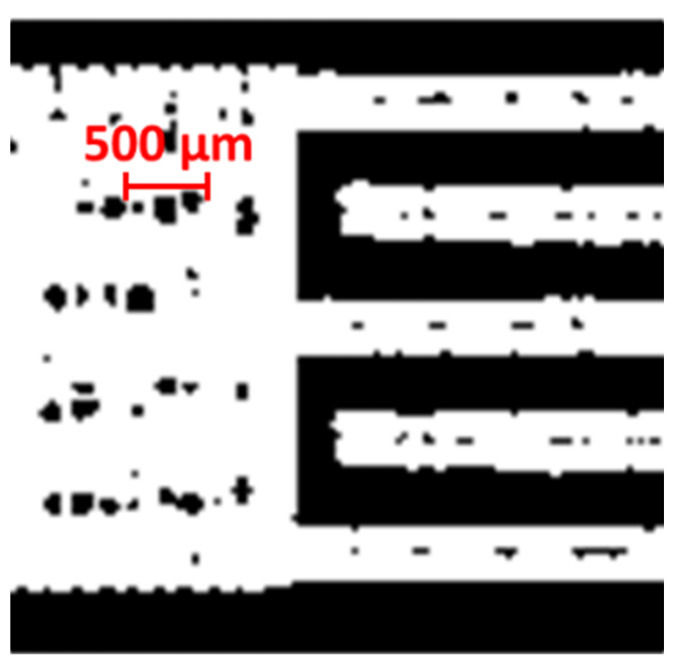
Detail of a binarized image of a sample including internal holes, which are the black spots in the white comb shapes (Δ*x* = 140 mm, Δ*y* = 170 mm, *v*_p_ = 10 mm/s, *T*_n_ = 35 °C).

In the MATLAB code, classical image process functions acquire features of each printed sample, binarizing (Figure 4) and labeling identified blobs. The reference geometry is composed by only two distinct blobs, corresponding to the two comb shapes that are spaced by 300 µm along their whole length. Therefore, images presenting more than two blobs contain isolated or disconnected shapes, which are removed from the analyzed image. Based on the obtained image, the Euclidean distance between the two blobs is calculated for each pixel on the blob contour. The code calculates the mean value, standard deviation, and minimum value of the whole set of measured distances (*μ*_gap_, *σ*_gap_ and *min*_gap_, respectively), excluding the distances exceeding a certain threshold (set to 500 µm). The gap length (*l*_gap_) corresponds to the number of pixels for which a gap distance is calculated, converted to millimeters. Due to the distance threshold, finger disconnections from the side rectangles or finger interruptions in random spots do not affect the calculation of *μ*_gap_ and *σ*_gap_, but reflect on the calculation of *l*_gap_.

## 3. Results and Discussion

Suitable models were analyzed to study the effect of the factors listed in Table 1 on the mean value (*μ*_gap_) and standard deviation of the gap (*σ*_gap_).

**Figure 5:**
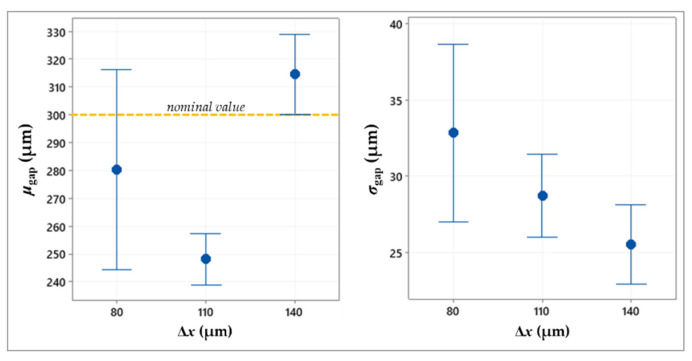
Interval plot of the mean value and standard deviation of the gap against drop spacing along the x-axis.

**Figure 6:**
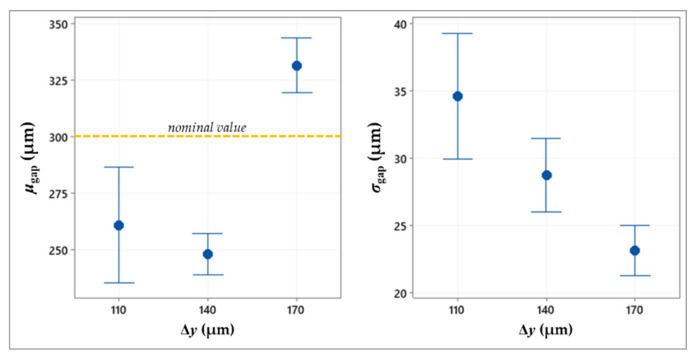
Interval plot of the mean value and standard deviation of the gap against drop spacing along the y-axis.

**Figure 7:**
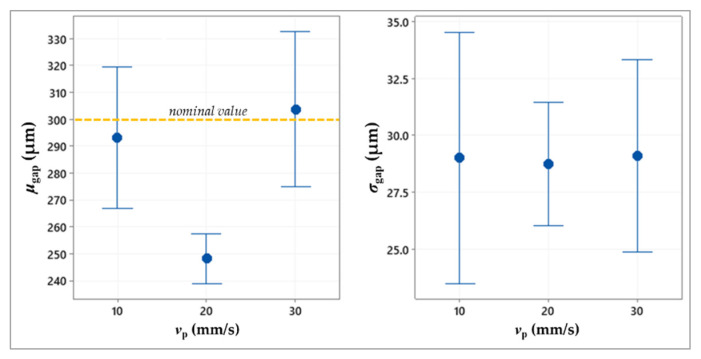
Interval plot of the mean value and standard deviation of the gap against the printing speed.

**Figure 8:**
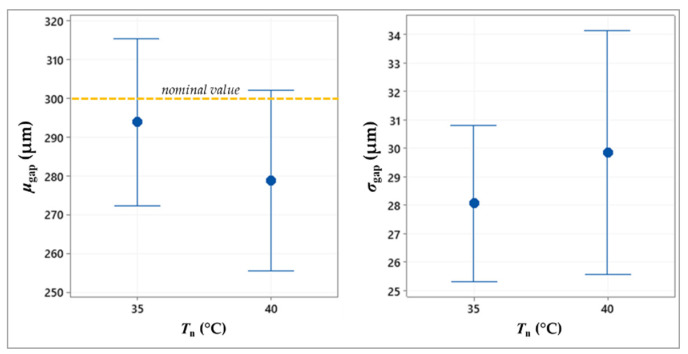
Interval plot of the mean value and standard deviation of the gap against the nozzle temperature.

Table 2 summarizes the ANOVA results, showing the statistically significant factors, while the plots in Figure 5, Figure 6, Figure 7 and Figure 8 depict the results related to the *μ*_gap_ and *σ*_gap_ for each factor.

Based on the ANOVA results resumed in Table 2, both responses are affected by the drop spacing along the x-axis and y-axis, respectively Δ*x* and Δ*y*. As both factors increase, the mean size of the gap shows values that are higher and closer to the nominal value (300 μm), while the standard deviation decreases (Figure 5 and Figure 6).

**Figure 9:**
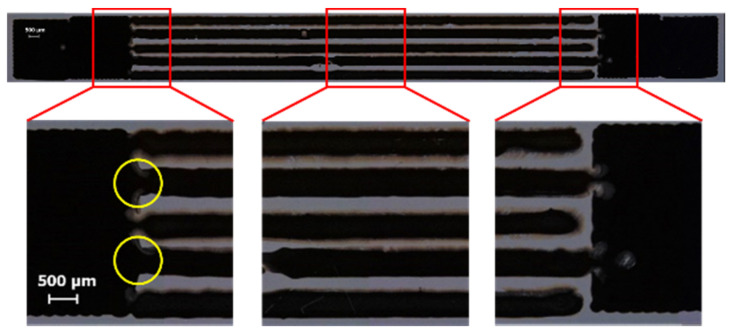
Example of printed sample with interconnected comb shapes (Δ*x* = 80 μm, Δ*y* = 110 μm, *v*_p_ = 10 mm/s, *T*_n_ = 40 °C).

Regardless of the direction, a higher spacing between the subsequent drops reduces the drop overlapping and, thus, the spreading of excess ink. Therefore, this allows obtaining lines that are thinner and more regular, helping to respect the target size and shape of the gap (*σ*_gap_ = 25.5 ± 4.9 μm at Δ*x* = 140 μm and *σ*_gap_ = 23.2 ± 3.4 μm at Δ*y* = 170 μm, as shown in Figure 5 and Figure 6). Conversely, lower spacing leads to undesired ink exceedances causing non-homogeneous boundaries, with consequent reduction of the gap mean value and increase in the gap standard deviation (e.g., in Figure 9).

The ANOVA results (Table 2) point out that the printing speed (*v*_p_) does not affect the two responses. This means that at the tested values of the printhead translational speed along the x-axis, the viscous friction forces acting on the drop after its ejection from the nozzle tip do not significantly influence the drop flight and, thus, do not cause drop instability. Furthermore, since *v*_p_ directly determines the jetting frequency, which is obtained as the ratio *v*_p_/Δ*x*, it can be stated that the investigated speed values also do not significantly modify the dynamic response of the fluid during the drop formation. Therefore, the tested *v*_p_ values belong to a feasible range for the investigated application. 

Based on the ANOVA results (Table 2), the nozzle temperature (*T*_n_) affects the mean size of the gap, whose value decreases as the temperature increase (Figure 8). This is probably caused by the dependence of ink viscosity from the temperature, which results in a higher ink spreading on the substrate when the temperature increases.

The minimum value of the gap (*min*_gap_) and the gap length (*l*_gap_) proved to be helpful in identifying undesired conditions with respect to the nominal geometry.

**Figure 10:**
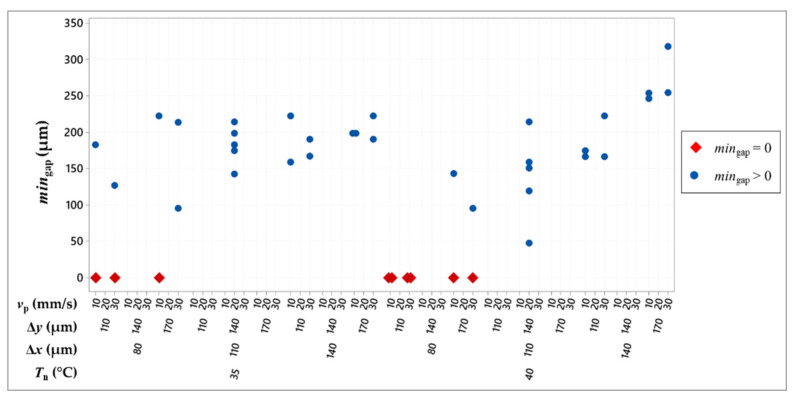
Individual value plot of the minimum value of the gap.

Figure 10 depicts the experimental results in terms of the minimum value of the gap. It should be noticed that the majority of the experimental conditions with Δ*x* = 80 μm resulted in a value of *min*_gap_ equal to zero (red diamonds in Figure 10), meaning that the comb shapes are interconnected, as shown by yellow circles in Figure 9, corrupting the geometry. Thus, all the process parameter combinations including a drop spacing along the x-axis equal to 80 μm are likely to be unsuitable.

**Figure 11:**
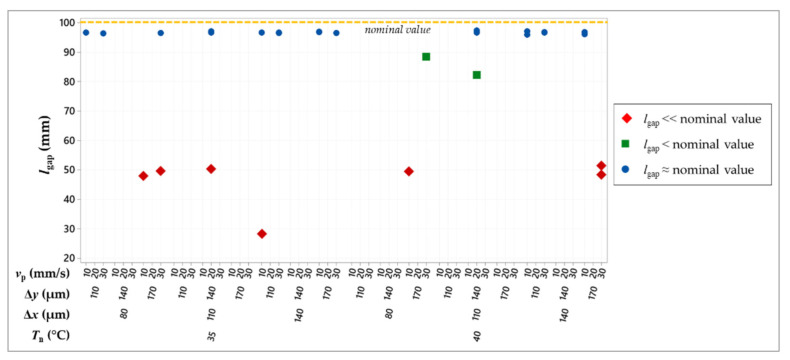
Individual value plot of gap length.

**Figure 12:**
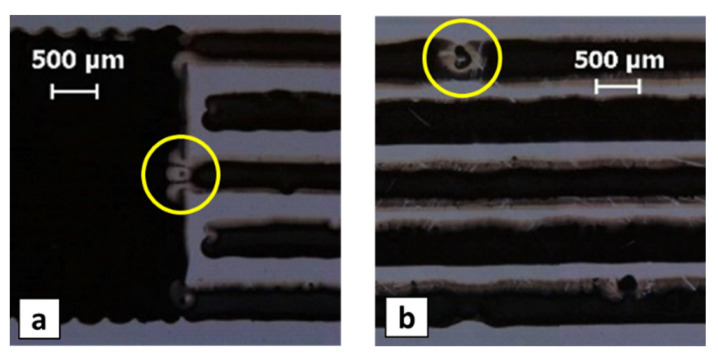
(**a**) Example of disconnection at the finger beginning (Δ*x* = 140 mm, Δ*y* = 170 mm, *v*_p_ = 30 mm/s, *T*_n_ = 35 °C); (**b**) Example of intermediate disconnection (Δ*x* = 110 mm, Δ*y* = 140 mm, *v*_p_ = 20 mm/s, *T*_n_ = 40 °C).

Figure 11 shows the *l*_gap_ values that were measured for the samples that do not present interconnections. A gap length that is much lower than the nominal value of 100 mm (red diamonds in Figure 11) implies that there are disconnections at the beginning of a finger (Figure 12a). A gap length that is slightly lower than the nominal value (green squares in Figure 11) means that there is a disconnection at some intermediate point of a finger (Figure 12b). The experimental data do not exhibit clear relationships between the process parameters and the disconnections, which are likely to be caused by random issues, such as dust or ink–substrate anomalous interaction, due to substrate defects or ink aggregates. 

## 4. Conclusions

This study investigated the application of the DoD inkjet printing technology to the manufacturing of a micrometer-scale representative geometry consisting of two interdigitated comb-shape electrodes. A suitable experimental design was studied to assess the influence of the spacing between subsequent drops, the printing speed, and the nozzle temperature on the printing process output. 

The experimental results showed that both the drop spacing along the x-axis and the drop spacing along the y-axis have an influence on the width and the regularity of the gap between the comb shapes. In particular, the spacing along the printing direction (x-axis) proved to be critical for avoiding interconnections between the comb shapes. Indeed, the drop spacing can influence the process output, since the distance among drops most dominantly affects the ink spreading on the substrate. Furthermore, the results pointed out that the nozzle temperature affects the gap mean value, probably by changing ink rheological properties such as viscosity and hence modifying the ink behavior. Eventually, the results showed that the printing speed does not influence the analyzed responses, suggesting that the tested values of this parameter belong to a feasible range for the investigated application. 

According to the experimental results, the parameter combination including Δ*x* = 140 µm, Δ*y* = 170 µm, and *T*_n_ = 35 °C is suggested to achieve a good accuracy of the printing output, that is, to obtain a geometry without interconnections or disconnections, and with a regular gap having a size close to the target one. The printing speed value can be selected throughout the investigated range, but using *v*_p_ = 30 mm/s would improve the process productivity, also ensuring a gap size close to the target one (Figure 7). 

The experimental results also showed that the process output was influenced by issues related to substrate damages or dust fibers. Therefore, performing the printing in a controlled environment could limit these issues and improve the quality of the manufactured geometries.

This work allowed creating a repeatable methodology for assessing the relationships between geometrical quantities and printing parameters, which can be extended to other ink–substrate couples. The developed analysis tools could also be used in a quality check procedure for batch-produced inkjet-printed shapes. To this end, the MATLAB code could be expanded with the evaluation of additional indices for quality assessment, such as the number of internal holes (i.e., empty spots in the printed shapes, as in Figure 4).

## Figures and Tables

**Table 1:** Experimental design summary.

Factor	Symbol	Levels	
		Low	High
X-axis spacing (μm)	Δ*x*	80	140
Y-axis spacing (μm)	Δ*y*	110	170
Printing speed (mm/s)	*v*_p_	10	30
Nozzle temperature (°C)	*T*_n_	35	40

**Table 2:** ANOVA *p*-values (bold = significant factor, confidence level α = 5%) for the analysis on the mean value and standard deviation of the gap.

Factors		*p*-Value	
		*μ*_gap_	*σ*_gap_
Main factors	Δ*x*	**0.000**	**0.001**
	Δ*y*	**0.000**	**0.000**
	*v*_p_	0.166	0.243
	*T*_n_	**0.030**	0.199
Interactions	Δx*Δ*y*	**0.000**	**0.043**
	Δx*v_p_	0.088	0.405
	Δx*T_n_	**0.043**	**0.003**
	Δy*v_p_	0.769	**0.021**
	Δy*T_n_	0.523	0.353
	*v*_p_*T_n_	0.721	0.201
